# Transduction of SIV-Specific TCR Genes into Rhesus Macaque CD8^+^ T Cells Conveys the Ability to Suppress SIV Replication

**DOI:** 10.1371/journal.pone.0023703

**Published:** 2011-08-23

**Authors:** Eugene V. Barsov, Matthew T. Trivett, Jacob T. Minang, Haosi Sun, Claes Ohlen, David E. Ott

**Affiliations:** AIDS and Cancer Virus Program, SAIC-Frederick Inc., National Cancer Institute at Frederick, Frederick, Maryland, United States of America; University of Pittsburgh, United States of America

## Abstract

**Background:**

The SIV/rhesus macaque model for HIV/AIDS is a powerful system for examining the contribution of T cells in the control of AIDS viruses. To better our understanding of CD8^+^ T-cell control of SIV replication in CD4^+^ T cells, we asked whether TCRs isolated from rhesus macaque CD8^+^ T-cell clones that exhibited varying abilities to suppress SIV replication could convey their suppressive properties to CD8^+^ T cells obtained from an uninfected/unvaccinated animal.

**Principal Findings:**

We transferred SIV-specific TCR genes isolated from rhesus macaque CD8^+^ T-cell clones with varying abilities to suppress SIV replication in vitro into CD8^+^ T cells obtained from an uninfected animal by retroviral transduction. After sorting and expansion, transduced CD8^+^ T-cell lines were obtained that specifically bound their cognate SIV tetramer. These cell lines displayed appropriate effector function and specificity, expressing intracellular IFNγ upon peptide stimulation. Importantly, the SIV suppression properties of the transduced cell lines mirrored those of the original TCR donor clones: cell lines expressing TCRs transferred from highly suppressive clones effectively reduced wild-type SIV replication, while expression of a non-suppressing TCR failed to reduce the spread of virus. However, all TCRs were able to suppress the replication of an SIV mutant that did not downregulate MHC-I, recapitulating the properties of their donor clones.

**Conclusions:**

Our results show that antigen-specific SIV suppression can be transferred between allogenic T cells simply by TCR gene transfer. This advance provides a platform for examining the contributions of TCRs versus the intrinsic effector characteristics of T-cell clones in virus suppression. Additionally, this approach can be applied to develop non-human primate models to evaluate adoptive T-cell transfer therapy for AIDS and other diseases.

## Introduction

Due to the central role of T lymphocytes in the cellular immune response, adoptive immunotherapy using autologous T cells is being evaluated in cancer treatment trials and as a means to suppress opportunistic virus outbreaks that occur in hematopoietic stem cell transplant patients [1], [2], [3], [4], [5], [6], [7], [8], [9], [10], [11], [12], [13]. Of the key factors in this approach, the isolation of T cells with the appropriate antigen specificity and robust effector functions is paramount. These requirements can be met by transferring highly effective TCR α/β chain gene pairs from donor antigen-specific T cells into recipient CD8^+^ T cells, thereby reprogramming them to display the antigen specificity of the donor cell [14], [15], [16], [17], [18], [19], [20], [21], [22], [23], [24], [25], [26]. Indeed, TCR-engineered autologous T cells have recently been successfully used in human clinical trials to treat melanoma [6], [27], [28], [29], [30], [31], [32].

Drug-based anti-HIV therapies are the clinically relevant tool against AIDS, yet emerging drug resistance remains a practical concern. While immune-based therapies hold great theoretical promise, practical treatments have not realized their potential due to an inability to understand the immune basis of immune control of HIV replication, the role CD8^+^ T cells play, the importance of the many effector functions, and the intrinsic difficulties with formulating and evaluating vaccines against HIV/AIDS. Unlike the theory behind cancer T-cell immunotherapy, it is unclear whether simply supplying more HIV-specific CD8^+^ T cells would necessarily provide better control of virus replication due to shortcomings of the HIV-reactive T cells themselves or the seemingly inexhaustible ability of HIV to escape the immune response by mutation [33], [34], [35], [36], [37], [38], [39], [40], [41], [42], [43], [44], [45], [46], [47]. Thus, while one of the basic problems in cancer therapy is a paucity of anti-tumor CD8^+^ T cells, one problem with T-cell-mediated control of HIV appears to be that while a broad CD8^+^ T-cell repertoire is generated, only a few of these T cells are effective at suppressing viral replication. Indeed, our prior studies of CD8^+^ T cells from either vaccinated or infected rhesus macaques found that actually only a fraction of the SIV-specific clones isolated could effectively suppress wild-type SIV in vitro even though all had similar IFNγ and degranulation properties [48].

One roadblock to our understanding of CD8^+^ T cell-mediated control of HIV/SIV is the lack of a suitable experimental in vivo model to answer these questions. Based on the intense interest and promise of adoptive transfer of TCR-engineered T cells for effective cancer therapy, we decided to apply this approach to the SIV/rhesus macaque model. Previous studies have transferred a single HIV-1 specific TCR between human CD8^+^ T cells and demonstrated the ability of the transduced cells to suppress HIV-1 replication [49], [50]. Here, we extended this approach to the rhesus macaque system, transferring three different TCRs cloned from SIV-specific CD8^+^ T cells into PBMC from an uninfected macaque. The resulting three transduced CD8^+^ T-cell lines stably expressed the transferred TCRs of the donors as measured by specific tetramer binding, exhibited CTL effector functions, and suppressed SIV replication in vitro to similar extent as the original donor clones, demonstrating effective transfer of TCR function to T cells from a SIV-naive animal.

## Results

### Transfer of SIV-specific TCRs into primary rhesus macaque T cells

**Figure 1:**
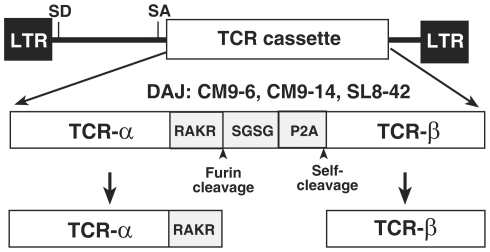
Diagram of TCR expressing retroviral vector and the mature TCR chains. The MSGV1 murine retroviral vectors sequences are displayed as black boxes and lines while the TCR expression cassette is in white. The fine structure of the TCR chain fusion cassette is presented below the vector with the different MamuA*01-restricted TCRs molecularly-cloned from DAJ T-cell clones that were inserted into the vectors indicated above the cassette. The sequences separating the TCR genes, the furin recognition sequence, KAKR, the S-G-S-G spacer, and the P2A fowl pox self-cleaving peptide, are shaded gray. The furin cleavage site and the P2A self-cleavage site are indicated below the cassette with arrows. The mature α and β chains produced by this vector are displayed at the bottom of the figure.

To transfer SIV-specific TCRs, we produced three TCR expressing vectors using TCR α and β chain-coding sequences that were cDNA cloned from two CD8^+^ T cell clones specific for the SIV_mac 239_ CM9 peptide, clones CM9–6 and CM9–14, and one that recognizes the SIV_mac 239_ SL8 peptide, clone SL8–42, all isolated from the SIV_mac239_ -infected animal, DAJ [51]. TCR α and β chain-coding sequences were inserted into the pMSGV murine retroviral vector [19] as a continuous open reading frame with the TCR genes (Fig. 1) separated by a spacer consisting of a furin protease cleavage site, which liberates the α chain protein from the linker leaving a 4 amino acids at its C-terminus, a Ser-Gly-Ser-Gly linker, and the P2A peptide from fowlpox virus [52], [53], which allows for expression of the β chain free from the N-terminal spacer polypeptide. Immunoblot analysis showed that this construct produced both α and β chains in the TCR-deficient human Jurkat J.RT3-T3.5 T cell line (data not shown).

**Figure 2:**
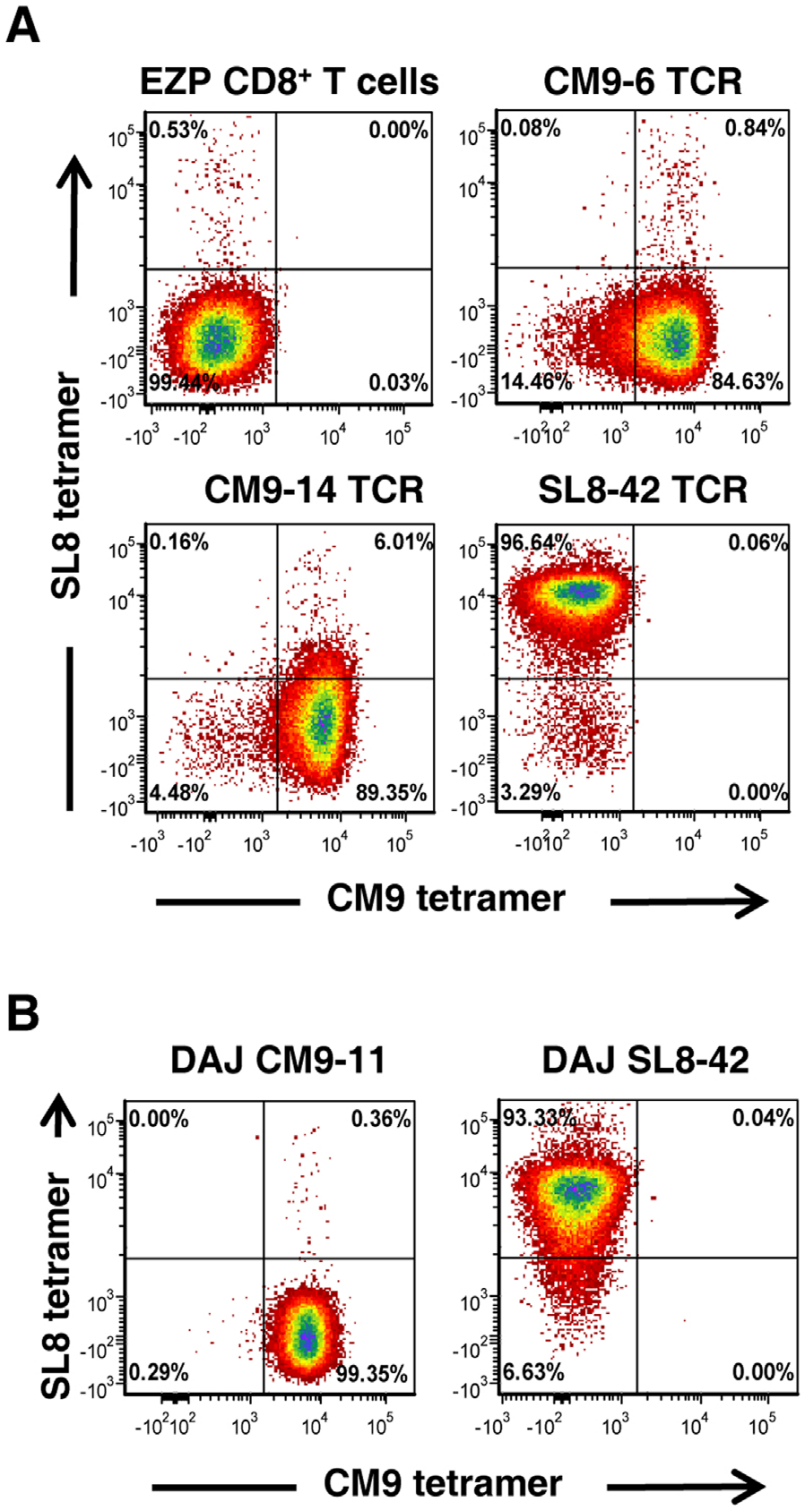
Flow cytometry analysis of transduced T cells. A, analysis of the TCR-transduced EZP cell lines for CM9 peptide/MHC tetramer and SL8 peptide/MHC tetramer is presented with that of the untransduced CD8^+^ control cell line from recipient animal EZP. B, tetramer analysis of two SIV-specific CTL clones isolated from donor animal DAJ is presented above tetramer-sorted TCR transduced CD8^+^ cell lines. The DAJ SL8–42 clone is the TCR gene donor for the SL8–42 TCR EZP cell line.

To transfer the donor Mamu A*01-restricted TCRs to primary rhesus macaque T cells, we transduced stimulated PBMC from a Mamu A*01-positive SIV-naïve monkey EZP with CM9–6, CM9–14, and SL8–42 TCR MSGV-based vectors. Flow cytometry analysis for tetramer binding by the transduced cells 48 hr post-transduction revealed that 5–9% of the CD8^+^ T cells bound their cognate tetramer with only a low background of staining cells detectable in an untransduced culture (data not shown). After expansion by anti-CD3 antibody stimulation, the cell cultures were sorted using tetramers and immunomagnetic beads, yielding cell lines that were between 84 and 96% positive for their cognate tetramer with low to no staining for an irrelevant tetramer (Fig 2). The density of TCR on the transduced T cells was similar to that on typical native clones (Fig. 2 and data not shown) and was maintained for the life-span of the cells (greater than 3 months, data not shown). Thus, we have stably transferred and expressed three SIV-specific TCRs isolated from DAJ into rhesus primary CD8^+^ T cells from EZP at levels similar to those observed on typical rhesus macaque T-cell clones.

### Transduced TCR cell lines exhibit specific effector function

**Figure 3:**
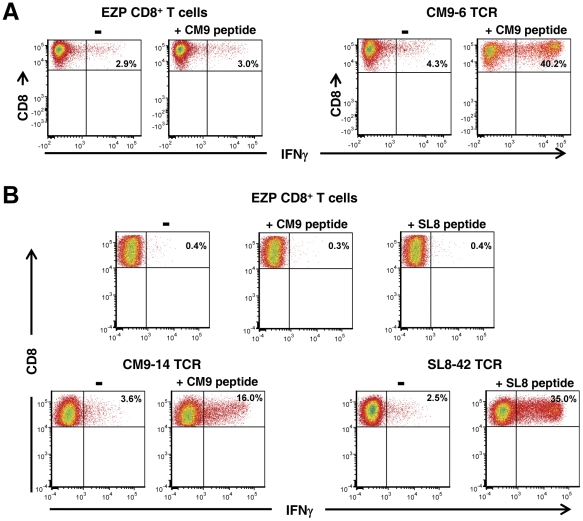
Intracellular IFNγ assay of TCR-transduced cell lines. Flow cytometry analysis of intracellular IFNγ production induced by the antigenic peptide is presented. Panel A, an assay of the CM9–6 TCR cell line stimulated with the CM9 peptide is displayed with its corresponding untransduced EZP CD8^+^ T-cell line control. Panel B, an assay of the CM9–14 TCR and SL8–42 TCR cell lines stimulated with either the CM9 or SL8 peptide is presented below their corresponding untransduced CD8^+^ T-cell controls. The stimulating peptide used is indicated above the respective plots.

To establish whether these cell lines exhibited antigen-induced effector properties, we assayed the cell lines for expression of intracellular IFNγ after stimulation with antigenic peptide, a hallmark of CTL effector function. Stimulation of the TCR cell lines with cognate peptide induced robust IFNγ responses (Fig. 3) and moderate CD107 degranulation (9–24%, data not shown) with only a low background of staining in the corresponding unstimulated control cultures, confirming the antigen-specific effector function of these TCR-transduced cells.

### Transduced T cell lines suppress SIV replication in vitro

**Figure 4:**
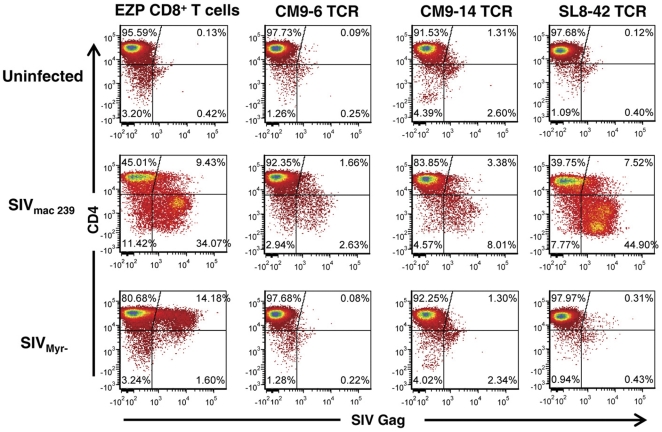
In *vitro* virus suppression assay of TCR-transduced cell lines. Flow cytometry analyses of mixed cultures consisting of effector CD8^+^ T-cell lines and a target autologous CD4^+^ T-cell clone that was untreated or exposed to either wild-type SIV_mac239_ or SIV_myr-_ are presented. Effectors are labeled above each column and targets are labeled at the left of each row. The effector CD8^+^ T cells in the co-cultures were stained with CellTrace Violet® and excluded from the analysis so that only the target cells were counted.

**Figure 5:**
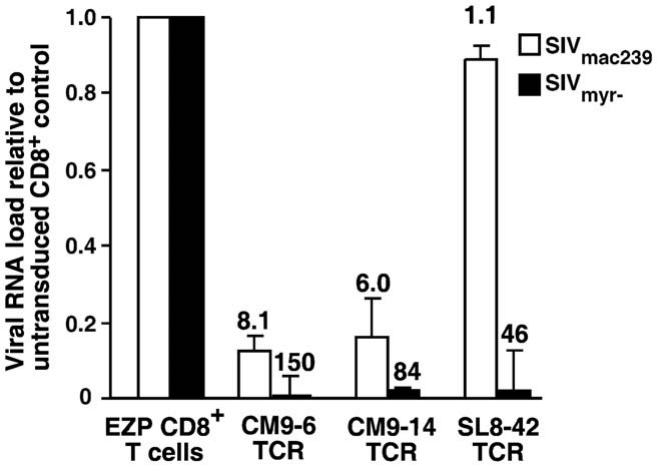
Suppression of viral RNA load. A graph of the viral load in the TCR-transduced T-cell mixed cultures relative to those containing untransduced CD8^+^ T cells is presented. The amount of viral suppression for each culture (viral load of the untransduced CD8^+^ cells co-culture divided by the load of anti-SIV effector cultures) is indicated above each bar. The average viral loads from duplicate independent PCR-based measurements of the untransduced CD8^+^ cell co-cultures were 6.6×10^8^ copies of SIV Gag per ml, SD 8.7×10^6^ for wild-type SIV_mac239_ and 3.8×10^8^ copies of SIV Gag per ml, SD 1.7×10^7^ for the myristylation mutant SIV_myr-_. Error bars represent standard deviations.

Since in vivo depletion experiments demonstrate that CD8^+^ T cells can suppress SIV infection in monkeys [54], [55], [56], [57], [58], [59], [60], [61], the CM9–14, CM9–6 and SL8–42 TCR cell lines were tested for their ability to suppress SIV. To model this function in vitro, we have previously developed a virus suppression assay that determines whether SIV-specific CD8^+^ T-cell clones can reduce the spread of SIV in autologous CD4^+^ T-cell clones exposed to SIV_mac239_ at relatively low multiplicities of infection [48], [51]. Because CD4^+^ downregulation in infected target cells makes it difficult to clearly distinguish them from effector cells, CD8^+^ T cells added to the co-cultures were stained with CellTrace Violet® and excluded from the analysis so that only the original CD4^+^ targets are analyzed. Co-cultures of infected EZP CD4^+^ T-cell cultures with untransduced EZP CD8^+^ T cells contained many SIV Gag staining cells (43% in this representative experiment) 7 days after infection. The rise in the proportion of infected cells was accompanied by a loss of surface CD4 expression on some cells (34%) due to downregulation by SIV protein expression (Fig. 4). In contrast to the untransduced CD8^+^ T cell negative control, co-culture of CM9–6 TCR cells with infected CD4^+^ T cells dramatically reduced the spread of the virus as evidenced by the presence of only 4% Gag positive target cells in the mixed culture (Fig. 4). This was also reflected in a 6-fold decrease in the supernatant viral RNA load 7-days post infection versus the control culture as measured by real-time RT-PCR (Fig. 5). The CM9–14 TCR cells were somewhat less effective at suppression than the CM9–6 TCR cells with 11% of the target cells being SIV Gag positive with a 4-fold suppression in viral RNA load. The SL8–42 TCR cells were ineffective at suppressing SIV spread with 52% of the target cells being SIV Gag positive (Fig. 4), essentially the same level of infection as the negative control with no noticeable effect on viral RNA load in the 7-day culture (Fig. 5) .

The results above essentially mirror those that we observed for the original DAJ donor clones: the CM9–6 (unpublished data) and CM9–14 [48] donor clones suppressed SIV_mac239_ while the SL8–42 DAJ clone failed to do so (unpublished data). However, we also had found that many clones, including the SL8–42 donor clone, while ineffective against the wild-type SIV_mac239_, were effective at suppressing the replication of an SIV Nef myristylation mutant, SIV_myr-_ [48], which does not down-regulate MHC-I on the surface of infected cells. To determine whether the SL8–42 TCR cells can suppress SIV in this less stringent system, we examined our panel of transduced cells using SIV_myr-_ in parallel with the wild-type suppression experiment. In agreement with data from the original DAJ donor SL8–42 clone, the SL8–42 TCR EZP cells were very effective in suppressing SIV_myr-_ replication when measured by both flow cytometry analysis (Fig. 4) and by viral load measurement 7-days post infection (Fig. 5). By flow cytometry analysis, the suppression was similar to that observed for the CM9–6 and CM9–14 TCR cells (Fig. 4). Correspondingly, the viral RNA loads in the SL8–42 TCR co-cultures revealed dramatically increased suppression of SIV_myr-_ viral loads compared to that observed with wild-type SIV (Fig. 5). Both CM9 TCR cell lines suppressed SIV_myr-_ viral RNA loads to a greater extent than that of wild-type SIV (Fig. 5), though the increase in suppression was less than that observed for SL8–42 TCR cells. These data show that while EZP CD8^+^ T cells expressing the SL8–42 TCR can suppress SIV replication, they can only do so in the absence of MHC-I down regulation, indicating a weaker avidity of the SL8–42 TCR.

## Discussion

The data presented here demonstrate the first transfer of antigen specificity via TCR transduction from T-cell clones isolated from an SIV-infected rhesus macaque into SIV naive T cells from an uninfected animal. Retroviral vector-mediated transduction of TCR genes resulted in a reprogramming of the recipient CD8^+^ T cells that conveyed SIV specific effector functions and virus suppression capabilities, i.e. the ability to curtail the spread of SIV in CD4^+^ target cells. While we present three intensively studied lines here, to date we have produced more than 20 TCR-transduced cell lines from EZP and two other animals with similar expression levels and properties (data not shown).

The transduced CD8^+^ T cells exhibited SIV epitope-specific CTL responses and virus suppression levels that were similar to their corresponding original TCR donor clones. Of the original CTL clones isolated from the DAJ animal, CM9–14 and CM9–6 were able to suppress wild-type SIV in our in vitro assay system while SL8–42 clone did not. Nevertheless, the DAJ SL8–42 clone could suppress SIV_myr-_ replication, indicating that, while this TCR was functional, it required higher levels of antigen density for anti-viral activity, reflecting a functional yet weaker TCR. The ability to faithfully recapitulate these SIV suppression properties by TCR transfer into the CD8^+^ T cells of another allogeneic MamuA*01 animal hints that this function is determined to a large extent by the nature of the TCR, rather than an intrinsic property of the T cell itself. It is important to note that we found essentially no difference in the TCR density on EZP CD8^+^ T cells transduced with the CM9 or SL8 TCRs from those on the original DAJ clones or other typical rhesus macaque T-cell clones (Fig. 2 and data not shown). However, an important caveat to this unexpected observation is that our TCR-transduced cell lines are populations of cells and not clones. Therefore it is possible that only a fraction of the transduced cells have strong suppressive function, thus are not representative of the population as a whole. Hence, the hypothesis suggested here requires additional investigation, including transducing T-cell clones with suppressive and nonsuppressive TCRs that recognize the same epitope to establish this observation.

This advance now allows for more in-depth experiments that extend our initial studies by examining the relative contributions of TCR and T cell to virus suppression and effector function. Transfers of TCR genes isolated from both suppressive and non-suppressive clones into characterized T-cell clones that have well defined properties are currently underway to build on our preliminary results presented here. Conversely, it is now feasible to place a well-defined TCR into different effector clones to address this question from the cellular side of CD8^+^ T-cell-mediated suppression.

To examine the role of CD8^+^ T cells in HIV-1 immunity, adoptive transfer experiments in the SIV model system have been employed to address questions about the qualitative and quantitative aspects of virus control. Indeed, other groups and we are investigating the potential of infusing large numbers of autologous anti-viral CD8^+^ CTL clones [62], [63]. These studies are hampered by difficulties in isolating and characterizing effective CD8^+^ CTL from virus-naïve vaccinees. By transducing CTL clones with TCRs isolated from CD8^+^ T cells exhibiting strong effector/suppressor properties, it may be possible to develop more potent anti-viral CTLs for evaluation in the rhesus/SIV system with the potential to apply the same approach to HIV/AIDS. Furthermore, placing an effective MHC restriction-matched TCR into a large and heterogeneous population of autologous T cells that contain an array of properties as accomplished here as opposed to relying on a single clone might also help overcome these difficulties. Therefore, the adaption of the TCR gene transfer system that has shown great promise in mice and human cancer studies to the SIV/rhesus macaque model provides for the direct assessment of anti-viral T-cell biology and a potential approach for antiviral therapy, especially to HIV/AIDS.

The ability to functionally transfer TCRs also provides for many exciting possibilities to manipulate the specificities of rare or unconventional T-cell phenotypes. Properties of T cells with different phenotypes or possessing important attributes, such as homing or persistence, could be comparatively studied with a defined TCR. Using this approach, the important relationship between the T-cell type, differentiation state, lifespan, or effector properties and the establishment of virus control/protection could be examined in adoptive immunotherapy experiments.

Despite years of study, the choice for the most reliable marker for highly effective functional anti-viral CD8^+^ T cells is not clear. One routinely used property is the induction of IFNγ upon peptide stimulation. However, the differences in wild-type SIV suppression seen here were not predicted by the level of IFNγ induction; all cell lines had similar induction profiles. We and others have previously observed a similar lack of correlation between viral suppression and detectable peptide titers that induce effector functions in vitro [48], [64], [65], [66]. Thus, our results support the conclusion that the widely used IFNγ induction marker does not necessarily predict anti-viral function and underscore the importance of testing for viral suppression to demonstrate bona fide anti-viral activity.

One complication to introducing cloned TCRs into CD8^+^ T cells that already express α/β TCR complexes is the prospect of forming hybrid dimers between the exogenous and endogenous chains [25], [67], [68], [69]. While we do not know to what extent hybrid complexes are being formed in our TCR-transduced CD8^+^ T cells, the T cells that we isolated by sorting the transduced populations for high tetramer binding had similar receptor densities to those of typical SIV specific CD8^+^ T-cell clones. Thus, cells forming large amounts of hybrid TCRs may not have been selected for our study. Nevertheless, the formation of hybrid dimers does not preclude the formation of functional exogenous TCR dimers as expressed by our vectors. In addition to inhibiting the formation of functional exogenous TCR complexes, in a therapeutic setting these hybrid α/β dimers could have unpredictable autoimmune specificities [70], [71]. While this has not been reported in cancer patients in TCR transfer trials, it is a concern for this approach in general. The adaption of the TCR transfer system to rhesus macaques presents an opportunity to examine the impact of hybrid TCR formation on long-term adoptive therapy in the monkey model with potentially important applications for TCR transfer therapy in the clinic.

Taken together, our results provide an important experimental tool for assessing and understanding T-cell function, especially SIV suppression. The application of TCR transfer to the rhesus macaque system could also make this important animal model even more useful for studies of other diseases. Current experiments are underway to answer fundamental and practical questions for immune control of SIV in rhesus macaques with the goal of developing a similar AIDS therapy in humans.

## Materials and Methods

### Ethical Treatment of Animals

Adult rhesus macaques (*Macaca mulatta*) were housed at the NIH-Bethesda primate animal facility that is accredited by the Association for the Assessment and Accreditation of Laboratory Animal Care International and under an approved OLAW Assurance #A4149-01. Research was conducted in compliance with the Animal Welfare Act and other US federal statutes and regulations relating to animals and experiments involving animals, and adhered to principles stated in the Guide for the Care and Use of Laboratory Animals, National Research Council, 1996 and under a protocol approved by the National Institutes of Health Intramural Institutional Animal Care and Use Committee, approval AVP-022. All steps were taken to ameliorate the welfare and to avoid the suffering of the animals in accordance with the “Weatherall report for the use of non-human primates” recommendations. Animals were housed either socially or in adjoining individual primate cages allowing social interactions, under controlled conditions of humidity, temperature and light (12-hour light/12-hour dark cycles). Food and water were available *ad libitum*. Animals were fed commercial monkey chow and treats by trained personnel. Environmental enrichment consisted of commercial toys. Blood draws were conducted under sedation by trained personnel under the supervision of veterinarians.

### Primary SIV-specificCD8^+^ T-cell clones

CM9-specific CTL clones were isolated by limited dilution cloning of CD8^+^ T cells from an SIV_mac_239 infected Mamu-A*01 positive Indian rhesus macaque, *Macaca mulatta*, as previously described [72]. Cell cultures were expanded by anti-CD3 antibody (BD Biosciences, Franklin Lakes, NJ) stimulation and cultured in RPMI 1640 medium supplemented with 10% FBS and human IL-2 (100 IU/ml). All culture media were obtained from Invitrogen Inc. (Carlsbad, CA). CD8^+^ T-cell clones CM9–14 [48], SL8–42 [51] and CM9–6 (unpublished) were selected for TCR gene isolation and retroviral vector construction.

### Cloning of TCR α and β chain cDNAs

TCR α and β chain coding sequences were isolated from the CD8^+^ T-cell clones using established cDNA cloning procedures. Briefly, total RNA was isolated from the cells by RNeasy Mini Kit (Qiagen, Valencia, CA), cDNAs were synthesized using an oligo-dT primer, and full-length α and β chain sequences were PCR-amplified by using a SMARTer RACE cDNA Amplification Kit (Clontech, Mountain View, CA). Full-length α chains were amplified by using the Universal Primer A Mix and either a rhesus macaque α or β chain constant region-specific primer. PCR products were cloned into the PCR XL-TOPO plasmid using TOPO XL PCR® Cloning Kit (Invitrogen, Carlsbad, CA) and the inserts were analyzed by DNA sequencing. Sequence analysis and alignments were performed using Vector NTI software (Invitrogen, Carlsbad, CA) and matched against the GenBank database. The TCR gene sequences presented here have been deposited in GenBank http://www.ncbi.nlm.nih.gov/sites/gquery (CM9–6 α chain, No. HQ622178, CM9–6 β chain, No. HQ622179, CM9–14 α chain, No. HQ622176, CM9-14 β chain, No. HQ622177, SL8–42 α chain, No. HQ622176, and SL8–42 β chain, No. HQ622177).

### TCR-expressing retroviral vectors

All DNA vector plasmids were constructed in the MSGV1 retroviral vector (kind gift of Richard Morgan National Cancer Institute, NIH, Bethesda, MD) [19], into which the CM9–6, CM9–14, and SL8–42 TCR α and β chain coding sequences were inserted as PCR-generated tandem open-reading frames with an intervening protein sequences consisting of: a furin protease recognition site, Ser-Gly-Ser-Gly, P2A fowlpox peptide linker (Fig. 1). The basic design of the PCR primers was to amplify both chains and then fuse them together by overlap extension. A 5′α sense primer with a linker/3′ α-anti-sense primer was used to amplify the α chain to produce a 5′ half of the TCR fusion and a linker/5′ β-sense primer with a 3′ β antisense was used to amplify the 3′ half. The two halves were then mixed and amplified with 5′α sense and 3′ β antisense primers which were designed with flanking Pci I and Not I sites, respectively, for cloning into the MSGV1 plasmid, thereby generating TCR CM9–6, TCR CM9–14, and SL8–42. All amplified regions were sequenced to ensure no PCR-introduced mutations.

The CM9–14 TCR construct was modified to contain additional cysteines to promote endogenous α/β dimer formation through an ectopic disulfide bond by mutating the α chain constant region Thr_48_ and the β chain constant region Ser_57_ to Cys by using site-directed mutagenesis with QuickChange® Multi Site Directed Mutagenesis Kit (Agilent Technologies, Santa Clara, CA), and the codon replacement was confirmed by sequencing.

### Generation of retroviral vector stocks

To produce the TCR retroviral vectors, the TCR plasmid constructs were co-transfected with a gibbon ape leukemia virus envelope expression construct (a kind gift of Paula Cannon University of Southern California, Los Angeles, CA) at a ratio of 8∶1 wt/wt into GP2-293 cells (Clontech, Mountain View, CA) by using Lipofectamine 2000 (Invitrogen, Carlsbad, CA), and the cells were cultured at 37°C. Vector-containing culture supernatants were harvested at 48 and 72 hours post transfection, clarified by low-speed centrifugation, buffered by 10 mM Hepes, and used for transductions. GP2-293 cells were maintained DMEM supplemented with 10% FBS, penicillin (100 U/ml), and streptomycin (100 µg/ml).

### Generation of TCR-transduced rhesus macaque T-cell lines

Freshly isolated PBMC from rhesus macaque EZP were stimulated by anti-CD3 simulation and then transduced by TCR-expressing retroviral vectors in the presence of RetroNectin® (TaKaRA Bio, Madison, WI). Briefly, RetroNectin® was diluted in PBS (Invitrogen) to 20 µg/ml and adsorbed onto the wells of 12 well tissue culture plates (Corning, Inc., Corning, NY) for two hours at room temperature. The wells were then washed with PBS, blocked with 2% BSA solution in PBS. Two ml of retroviral vector stock were added to each well and the plates were spun down at 3,000 rpm at 30°C for two hours. The target cells were resuspended in the medium (RPMI 1640 with 10% FBS, supplemented with 100 IU/ml of IL-2) at 1×10^6^ cells/ml. The wells were washed once with 2% BSA in PBS, the cells (1–2×10^6^ cells per well) were added and the plates were centrifuged at 1,000 rpm (30°C) for 40 min. The plates were placed at 37°C (5% CO_2_) and the cells were cultured for 48–96 hours before tetramer binding by flow cytometry analysis using CM9 peptide/MHC tetramer (Beckman Coulter, Brea, CA) or SL8–42 peptide/MHC tetramer (NIH Tetramer Core Facility, Atlanta GA). Cultures containing tetramer positive cells (at least 3% of the population) were expanded by anti-CD3 antibody stimulation as previously described [72] and then sorted for tetramer binding by paramagnetic microbeads. TCR–expressing cells were labeled with either CM9 or SL8–42 tetramer conjugated with PE, then washed with PBS, bound to anti-PE Microbeads (Miltenyi Biotech, Auburn, CA), and separated by using magnetic columns (Miltenyi Biotech, Auburn, CA). Isolated T cells were expanded as described above for the primary T-cell clones.

### Flow cytometry analysis of TCR-transduced T- cell lines

1–2×10^6^ TCR-transduced cells were harvested and washed once in 2 ml of D-PBS, resuspended and stained with a cocktail containing either CM9 peptide/MHC tetramer, or SL8–42 peptide/MHC tetramer, and CD3, CD8, CD45 antibodies (BD Biosciences) and D-PBS in a final volume of 100 µl. Cells were incubated 30 minutes at room temperature in the dark, washed once in 4 ml of D-PBS and then analyzed immediately by an LSRII flow cytometer (BD Biosciences) Data analysis was performed using FCS Express (De Novo Software, Los Angeles, CA).

### Intracellular IFNγ assay

Intracellular IFNγ production was measured as previously described [72] with modifications as follows. For each sample, 10^6^ cells from EZP CD8^+^ T-cell lines were labeled as peptide presenters with CellTrace Violet® (Molecular Probes/Invitrogen, Eugene, OR) according to the manufacturer's procedure and then pulsed for 30 minutes with 2 µg of either CM9 or SL8 peptide (SynPep, Dublin, CA) followed by two PBS washes before addition of 10^6^ TCR-transduced T cells. The rest of the analysis was carried out using our previously published IFNγ assay on an LSRII flow cytometer. The pulsed CD8^+^ T-cell presenters were excluded from the analysis by virtue of their staining by CellTrace Violet®.

### SIV Suppression assay

Target CD4^+^ T cells were activated with plate-bound anti-CD3 antibody in the presence of IL-2 (50 IU/ml) for 24 hours and infected with either SIV_mac239_ or a Nef myristylation mutant SIV_myr-_ [48] by incubating virus stocks (∼1×10^9^ viral RNA copies Eq/ml in a volume of 250 µl) with 2 µl of Viromag paramagnetic beads (OzBiosciences, Marseille, France) for 2–3 hours before addition to 1×10^6^ CD4^+^ T cells. CD4^+^ T cells were first labeled with CellTrace Violet®. Incubations were carried out on a magnetic plate at 37°C in a humidified atmosphere of 5% CO_2_. Virus-exposed CD4^+^ T cells were washed twice with PBS to remove residual non-incorporated virions before co-culture with effector CD8^+^ T cells as previously described. Both cells and virus were analyzed on day 7 post co-culture by flow cytometry and real-time RT-PCR, respectively, as previously described [51]. Our previous experiments demonstrated that proviral loads matched viral RNA loads in the mixed cultures so this redundant measurement was omitted in this study. CD4^+^ T cells were differentiated from the effector CD8^+^ T cells in flow cytometry analysis by staining with CellTrace Violet®.
