# Correction: Transduction of SIV-Specific TCR Genes into Rhesus Macaque CD8^+^ T Cells Conveys the Ability to Suppress SIV Replication

**DOI:** 10.1371/journal.pone.0195246

**Published:** 2018-03-28

**Authors:** Eugene V. Barsov, Matthew T. Trivett, Jacob T. Minang, Haosi Sun, Claes Ohlen, David E. Ott

There are errors in the final sentence of the section, “Cloning of TCR α and β chain cDNAs.” The accession numbers for the SL8–42 α chain and SL8–42 β chain were listed incorrectly. The correct sentence is as follows: The TCR gene sequences presented here have been deposited in GenBank http://www.ncbi.nlm.nih.gov/sites/gquery (CM9–6 α chain, No. HQ622178, CM9–6 β chain, No. HQ622179, CM9–14 α chain, No. HQ622176, CM9-14 β chain, No. HQ622177, SL8–42 α chain, No. HQ622180, and SL8–42 β chain, No. HQ622181).
